# Acclimation to water stress improves tolerance to heat and freezing in a common alpine grass

**DOI:** 10.1007/s00442-022-05245-1

**Published:** 2022-08-17

**Authors:** Emma E. Sumner, Virginia G. Williamson, Roslyn M. Gleadow, Tricia Wevill, Susanna E. Venn

**Affiliations:** 1 Centre for Integrative Ecology, School of Life and Environmental Sciences, Deakin University, Burwood, 3125 Australia; 2 School of Biological Sciences, Monash University, Clayton, 3800 Australia

**Keywords:** Acclimation, Alpine plants, Climate change, Drought, Heat, Stress, Thermotolerance

## Abstract

Alpine plants in Australia are increasingly exposed to more frequent drought and heatwaves, with significant consequences for physiological stress responses. Acclimation is a critical feature that allows plants to improve tolerance to environmental extremes by directly altering their physiology or morphology. Yet it is unclear how plant performance, tolerance, and recovery are affected when heat and water stress co-occur, and whether prior exposure affects responses to subsequent climate extremes. We grew a common alpine grass species under high or low watering treatments for three weeks before exposure to either none, one, or two heat stress events. We determined photosynthetic heat and freezing tolerance (LT_50_, mean temperature causing 50% irreversible damage to photosystem II) and growth. Physiological adjustments to low watering, including more negative water potentials and reduced growth, were also characterised by improved tolerance to high and low-temperature extremes. Shifts to higher heat tolerance were also evident with increasing exposure to heat stress events, though freezing tolerance was not affected. Acclimation effects were mostly short-term, however; prior exposure to heat and/or water stress had little to no effect on growth and thermal tolerance following the six-week recovery period. We conclude that rapid acclimation to water and heat stress that co-occur during summer enhances the capacity of alpine plants to tolerate increasingly frequent temperature extremes.

**Supplementary Information:**

The online version contains supplementary material available at 10.1007/s00442-022-05245-1.

## Introduction

Climate change is driving increases in the frequency, duration and intensity of extreme weather events such as heatwaves and drought (Cowan et al. 2014; Arias et al. 2021). Characterised by consecutive days of high temperatures above, for instance, the 90th percentile for maximum temperature (Perkins and Alexander 2013), heatwaves represent a major challenge for all plant species, but particularly those in high elevation ecosystems which are considered especially vulnerable to the effects of climate change due to their limited spatial extent and a high degree of endemism (Pauli et al. 2012). In southern Australia, the hottest heatwaves are predicted to increase by ~ 3 °C by the end of the century under RCP 8.5 (high-emission scenario) (Cowan et al. 2014). Moreover, heatwaves are likely to occur simultaneously with drought (Arias et al. 2021), and combinations of two different stresses (i.e., water and heat stress) may have unique consequences for plant reproduction, growth, and survival (Mittler 2006; Suzuki et al. 2014). Physiological mechanisms are highly sensitive to thermal stress, hence the thermal tolerance of photosynthetic tissues can indicate the potential of plant species to tolerate and acclimate to an increasingly hotter climate (Orsenigo et al. 2014; Geange et al. 2021). Acclimation to combined heat and water stress may alter how alpine plants respond to the rapid temperature fluctuations that characterise alpine plant microclimates.

The thermal environment of alpine plants is inherently unstable. In the alpine zone, rapid fluctuations in daytime temperatures can contrast with freezing temperatures during clear nights (Körner and Hiltbrunner 2018). Moreover, plant leaves typically attain much higher temperatures than ambient air temperatures, due to the moderating effects of plant stature, topography, wind velocity, and solar irradiation (Körner and Hiltbrunner 2018). Under clear skies, leaf temperatures of short-statured alpine plants depart rapidly from air temperatures, often reaching approximately 10–26 °C higher (Salisbury and Spomer 1964; Sage and Sage 2002; Körner and Hiltbrunner 2018). In the Ecuadorian páramo, Ramsay (2001) observed that air trapping within the upper parts of grass tussocks saw leaf temperatures rise more than 5 °C above ambient air temperatures during the day. Leaf temperatures above 40 °C have also been recorded for brief periods (up to 20 min) in *Carex breviculmis* (Cyperaceae) in the Australian alpine zone (E. Sumner, unpublished data). Subsequent passing cloud, fog, or wind gusts can rapidly recouple plant leaves to ambient temperatures (Salisbury and Spomer 1964; Sage and Sage 2002; Körner 2003). Extreme temperatures can induce obvious damage to leaves and reproductive structures (Neuner 2014; Neuner et al. 2020), impair photosynthetic capacity (Filewod and Thomas 2014), and can result in reduced growth, and mortality (Marcante et al. 2012; French et al. 2019). Therefore, rapid thermotolerance acclimation is necessary to ensure survival under such challenging conditions.

Acclimation to high temperatures may alter the capacity of alpine plants to respond to the rapid temperature fluctuations that occur in the alpine environment. Exposure to non-lethal high temperatures (typically above 30 °C) is known to induce acclimation whereby physiological changes can improve the thermostability of Photosystem II (PSII) and thus reduce or mitigate plant cellular injuries caused by extreme temperatures (Schreiber and Berry 1977; Neuner et al. 2000; Buchner et al. 2017). Acclimation of photosynthetic heat tolerance to hotter growing conditions may provide plants with a greater buffer to the brief high-temperature extremes that can occur under high solar irradiation and calm conditions. Plants acclimating to a hotter climate, however, may simultaneously lose their tolerance to frost (Rixen et al. 2012). Triggered by seasonal changes to the photoperiod and to cooling temperatures, full frost acclimation can take several weeks to months in duration to develop (Franklin et al. 2014). By comparison, de-acclimation (a reduction in the levels of freezing tolerance attained via the previous acclimation), can occur rapidly upon exposure to increases in ambient temperature (Franklin et al. 2014) and can leave plants vulnerable to episodic frost events during the growing season (Inouye 2008; Rixen et al. 2012).

Water stress and extreme temperatures commonly occur simultaneously under natural conditions (Buchner et al. 2017). During winter, drought due to frozen soil, and low-temperature extremes commonly combine, while in summer, drought periods can co-occur with heatwaves (Mayr et al. 2006; Suzuki et al. 2014). Plant water stress is a major determinant of thermal tolerance, as it can significantly alter the ability of plants to respond to temperature extremes (Kong and Henry 2019a). This is because there is some overlap between cellular responses to different forms of environmental stress (Suzuki et al. 2014). For instance, as both water stress and freezing affect the water relations of plants at a cellular and whole-plant level, acclimation to one stress may result in improved tolerance to the other (Beck et al. 2007). Indeed, higher freezing resistance has been reported for plants from dry growing conditions (Sierra-Almeida and Cavieres 2010), while experimental drought has been shown to cause smaller reductions in biomass amongst frost-tolerant progeny of Norway spruce (Blödner et al. 2005). The relationship between water stress and heat, however, is likely to be species-specific. In some plant species, water stress can either improve or reduce the heat tolerance of PSII (Buchner and Neuner 2003). As water stress alone can impair growth and photosynthesis, simultaneous abiotic stress factors such as water stress and heat may lead to cumulative damage, much more severe than the effects of each stressor in isolation (Nicolas et al. 1984; Orsenigo et al. 2014). Swiss alpine grassland communities showed reduced above-ground biomass growth when heatwaves coincided with water stress (De Boeck et al. 2016), for instance; an effect that was still evident after two years of monitoring (De Boeck et al. 2018).

Given the ongoing increases in the frequency, intensity, and duration of climate extremes including heatwaves and drought in high elevation ecosystems, a better understanding of how plants respond to simultaneous stressors is necessary to predict vegetation and ecosystem change. Moreover, due to the naturally fluctuating thermal microclimate experienced by alpine plants, it is also essential to assess responses to the full breadth of plant thermal tolerance. Here, we assessed plant responses (heat tolerance, freezing tolerance, water status and growth) to the factors of heat and water stress, along with their interactions.

Specifically, we asked:

1. Does exposure to combined heat and water stress alter photosynthetic heat and freezing tolerance, water status and growth?
2. Is heat acclimation, and any associated improvement in heat tolerance, antagonistic with freezing tolerance?
3. Does leaf heat tolerance increase with exposure to heat stress events?
4. Does prior stress exposure alter photosynthetic heat and freezing tolerance and growth after a recovery period?

To address these research questions, we grew a common Australian alpine grass under high and low water treatments and used the controlled environment of a glasshouse to provide two heat stress events. Subsequently, photosynthetic heat and freezing tolerance were estimated to determine the capacity of plants to tolerate brief high-temperature extremes and freezing events.

## Materials and methods

### Plant material and experimental design

*Poa hothamensi*s var. *hothamensis* N.G. Walsh, (hereafter referred to as *P. hothamensis*) is generally between 20 and 30 cm in height but can grow to a height of 90 cm under favourable conditions in sub-alpine environments. *P. hothamensis* is locally common in sub-alpine and alpine shrublands above 1200 m asl. The leaves of *P. hothamensis* are distinctly flat-bladed and it flowers from December to February in the Austral summer. Seed was provided by the Victorian Alps Nursery, collected from the Mt Hotham area in January 2016 from a grassland (36°59′43″S, 147°10′5″E) situated at 1650 m asl and stored at 7 °C. In June 2020, seed was surface sown onto Scotts Osmocote seed and cutting mix, and subsequent germinants were grown with Scotts Osmocote native premium potting mix in individual 100 × 60 mm pots for 14 weeks over winter and spring under ambient conditions in Melbourne, Australia (37°50′27″S, 144°56′47″E). In November, plants were moved to a climate-controlled glasshouse and grown under summer alpine conditions (day/night cycle of 18 °C/7 °C). Prior to experimental treatments, a visual assessment was made of root growth. There was no evidence of any of our replicates being root-bound, hence they were maintained in the original 100 mm × 60 mm pots for the duration of the experiment. At this stage, the tussocks were approximately 20–30 cm tall, with approximately 20 fully expanded leaves per tussock. The pots were well-watered for 7 days until the water stress treatment was initiated.

### Water and heat stress treatments

To quantify the individual and combined effects of water stress and heat stress on *P. hothamensis,* plants were randomly assigned to the two watering and heat stress treatments in a 2 × 3 factorial design. From mid-November, two watering treatments were initiated: plants were watered to either 100% (high) or 60% (low) pot capacity (PC); pot capacity being a measure analogous to field capacity (FC), which is the water content that a pot retains at saturation. To determine PC, all pots were watered to saturation by submerging in water overnight, covered to reduce evaporation, and then drained for 4 h the following day with subsequent individual pot mass defined as 100% PC. The ‘low’ watering treatment was initiated by withholding water until 60% maximum PC was reached. Plants in the high and low watering treatments were maintained at 100% and 60% PC, respectively, for three weeks by weighing the pots daily and rewatering to the target weight. Plants were moved three times between adjacent benches within the glasshouse during the treatment period to reduce potential glasshouse effects.

Following three weeks under high and low water treatments, plants were then subjected to either one heat stress event (*n* = 20), two heat stress events (*n* = 20), or no heat stress events (*n* = 20). During heat stress events, type T thermocouples (Model TC6-T, Onset, Bourne, USA) were attached to the undersides of leaf blades using Micropore surgical tape and leaf temperatures were recorded once every minute using thermocouple data loggers (Model UX120-014 M, Onset, Bourne, USA). We maintained high and low watering treatments between 0:900 and 17:00 by rewatering to the correct treatment weights every 2 h to mitigate higher evaporation during heat stress treatments.

Heat stress event day/night conditions were approximately 35 °C/25 °C and were based on ongoing increases to high-temperature maxima particularly in the hottest month (January) recorded from a weather station closest to the site of seed collection (Mount Hotham; 1849 m asl) where the highest maximum temperature recorded to date is 28.2 °C during a heatwave in January 2020 (Supplementary Materials Fig. S2, S3).

**Fig. 1:**
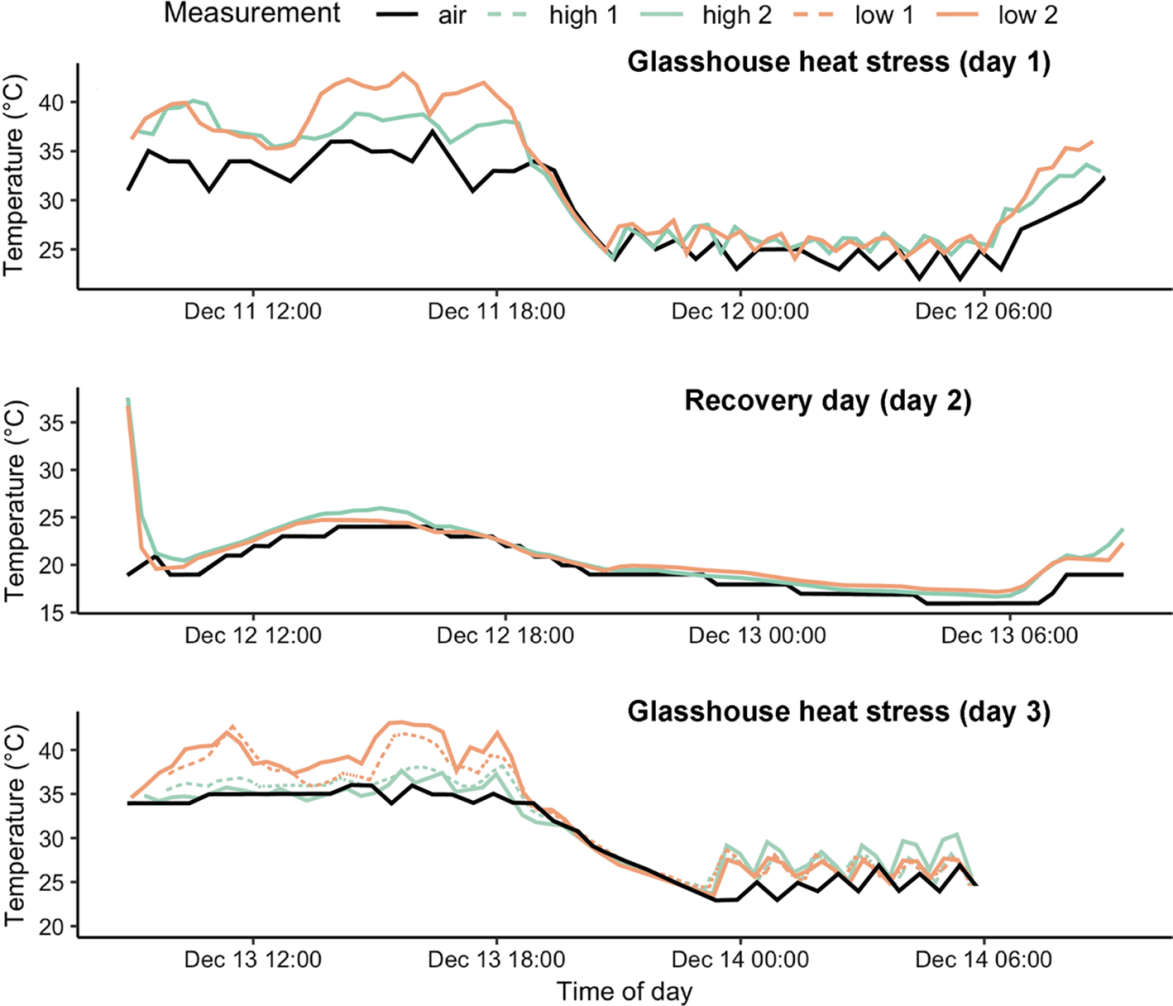
Temperature maxima during the glasshouse heat stress and recovery days. *Solid black line* air temperature; *solid green line* high watering treatment + 2 heat stress events; *dashed green line* high watering treatment + 1 heat stress event; *solid orange line* low watering treatment + 2 heat stress events; *dashed orange line* low watering treatment + 1 heat stress event. High watering treatment refers to *P. hothamensis* plants watered to 100% PC and low watering treatment refers to plants held at 60% PC. Leaf temperature maxima reflect the maximum value recorded amongst leaves of plant replicates (*n* = 4) during intervals of 20 min

The first heat stress event (day 1) lasted 24 h (09:00–09:00) and the maximum air temperature was 37 °C (Fig. 1). Plants were returned to alpine summer conditions (day 2) (day/night cycle of 18 °C/7 °C) for 21 h (09:00–06:00) with a maximum recorded air temperature of 24 °C. The second heat stress event (day 3) lasted 21 h (09:00–06:00) where the maximum air temperature was 36 °C, and after which plants were returned to control conditions on day 4 (called here the recovery period) for six weeks. During heat stress days, plants were moved from the control glasshouse to the pre-heated glasshouse at 9:00 where the air temperature was approximately 30 °C. Plant replicates in treatment groups receiving only one heat stress event were introduced to heat stress conditions during the second heat stress event (day 3). Physiological measurements were made at two timepoints: immediately after the treatment period (day 4) and again at the end of the experiment, after six weeks’ recovery, prior to being destructively harvested for biomass growth analysis (Supplementary Materials, Fig. S1).

### Determination of water status

Water status was measured as water potential (*Ψ*). At the first timepoint, to measure plant water status in relation to the experimental water stress and heat stress treatments, five individuals from each treatment were harvested to determine pre-dawn (*Ψ*_pd_) and midday (around 12 noon, accounting for daylight savings time) (*Ψ*_md_) water potentials. To ensure plant material was collected pre-dawn, astronomical dawn, when the sun is 18° below the horizon and the sky is completely dark, was determined from https://www.timeanddate.com/sun/australia/melbourne?month=12. Water potential was determined on healthy, fully expanded adult leaf blades (*n* = 5) immediately after excision using a pressure chamber (Model 1000, PMS Instrument Co., Albany, USA) fitted with a grass compression gland (PMS Instrument Co., Albany, USA). Pressure within the chamber was raised slowly (0.05 MPa s^−1^) to ensure the equilibrium of water throughout the plant and to reduce pressure-associated temperature changes (Tyree et al. 1978). Volumetric soil moisture was determined following water potential measurements using a soil ThetaProbe soil moisture sensor (Model ML3, Delta-T Devices, Cambridge, UK).

### Heat assay to determine photosynthetic heat tolerance

Leaf samples for the determination of photosynthetic heat tolerance and freezing tolerance were excised from the same replicates following *Ψ*_pd_ measurements. Fully expanded healthy leaves were sampled from each treatment replicate and placed into sealed plastic bags with a moist paper towel at room temperature (approximately 18–23 °C) in the dark until randomly selected for thermal tolerance assays. Both heat and freezing tolerance assays were initiated approximately 3 h after excision.

Three leaves from each plant replicate were treated with one of six hot temperature shock treatments (44 °C, 47 °C, 50 °C, 53 °C, 56 °C and a 22 °C control) using temperature-controlled hot water baths. From each replicate, leaf blades were randomly chosen from the sampling bags and placed onto moistened paper towel inside clear waterproof plastic pouches, which were then sealed to avoid dampening the samples in the water baths. The target temperature of each hot water bath was maintained using precision temperature immersion circulators typically used for *sous vide* low-temperature cooking (Model KASTKSOVIDB: Kogan, Melbourne, Australia). Treatment temperatures were verified using type T beaded thermocouples positioned at the same depth at which plant samples were placed in the water baths and monitored throughout the heat treatment using thermocouple data loggers (Model: UX120-014 M Onset, Bourne, USA). Each immersion circulator was placed into a 70 cm × 30 cm metal tub, which was fitted with a metal rack 3 cm below the water surface for the placement of leaf blade samples. Light during heat exposure activates the production of protective pigments and has been shown to provide more realistic heat tolerance estimates (Krause et al. 2016). As such, throughout the entire treatment process, plant samples were exposed to Photosynthetically Active Radiation (PAR) of approximately 800 µmol m^−2^ s^−1^ using two 150 W full-spectrum LED grow lights suspended 9 cm above the surface of the water in each water bath. PAR was determined under the surface of the water to the depth at which samples were placed using a Quantum Sensor (Model LI-250A: LI-COR Inc, Lincoln, USA). Following Curtis et al. (2014), and based on field recorded duration of leaf temperatures of *Carex breviculmis* (Cyperaceae) > 40 °C (E. Sumner, unpublished data), we chose a 15 min heat exposure period to represent sudden short-term high leaf temperatures that occur during periods of still air and high solar irradiation in summer.

Heat tolerance assays were carried out using the following protocol: (1) samples were placed under control conditions (22 °C, 800 µmol m^−2^ s^−1^ PAR) for 15 min; (2) samples were removed from control conditions and immediately placed under treatment conditions (44 °C, 47 °C, 50°, 53 °C, or 56 °C with 800 µmol m^−2^ s^−1^ PAR) for 15 min; (3) samples were removed from treatment conditions and placed again under control conditions for 90 min; (4) samples were removed from control conditions and stored at room temperature (approximately 18–23 °C) in the dark for approximately 16 h to allow for partial recovery of PSII following Krause et al. (2010); (5) Maximum quantum efficiency of open PSII centres (*F*_V_/*F*_M_), the ratio of variable to maximum fluorescence, following Maxwell and Johnson (2000) was measured using an Imaging PAM (Model IMAG-MIN/B: Walz, Effeltrich, Germany).

### Freezing assay to determine photosynthetic freezing tolerance

Three leaves from each replicate were treated to one of six cold temperature treatments (0 °C, − 5 °C, − 10 °C, − 15 °C, − 20 °C and a 5 °C control) using portable 45 L compressor freezers (Model BCD-45L: Adventure Kings, NSW, Australia). Leaves were placed into separate zip-lock plastic bags with a moistened paper towel and the air was removed before sealing. Sample replicates were placed into the freezers that had been previously set to 5 °C for one hour, after which each freezer temperature was manually ramped down at a rate of 5 °C h^−1^ until the target temperature was reached. Freezer temperatures were monitored throughout the duration of freezing assays with digital thermometers and validated using iButton temperature loggers (Model TC: Thermochron, Baulkham Hills, Australia). Samples were held at their target temperatures for 8 h, chosen to reflect the typical length of freezing events reported for alpine areas in Australia (Bureau of Meteorology, 2022). After 8 h, freezers were switched off and allowed to return to room temperature which occurred at a rate of approximately 4 °C h^−1^. Plant material was kept inside the freezers at room temperature to recover in the dark for approximately 72 h, after which *F*_V_/*F*_M_ was measured using an Imaging PAM (Model IMAG-MIN/B: Walz, Effeltrich, Germany), as described above.

### Thermal tolerance after the recovery period

Following the first thermal tolerance assays, the drought treatment was ceased and the remaining plants from each treatment were re-potted into 100 mm × 100 mm pots and subsequently grown under alpine summer conditions (day/night cycle of 18 °C/7 °C) with twice daily irrigation using automated drippers. After six weeks, replicates from each treatment (*n* = 5) were destructively harvested for the determination of photosynthetic thermal tolerance following the heat and freezing assay protocols previously described. Volumetric soil moisture was also measured at the time of harvest using a ThetaProbe soil moisture sensor (Model ML3: Delta-T Devices, Cambridge, UK).

### Plant growth analysis

Following heat stress treatments and recovery periods, replicates (*n* = 5) were destructively harvested and separated into above-ground phytomass (living biomass and dead necromass) and root biomass. Roots were gently washed free from the potting mix with water. All biomass samples were then dried at 60 °C for 48 h and weighed to determine above and below-ground growth responses, the ratio of root biomass to shoot biomass (*R*/*S*), and total dry biomass (TDB) which is the total dry weight of phytomass and root biomass.

### Statistical analyses

Data analyses were performed using R software (version 4.0.5, R core team 2022). To obtain LT_50_ values for both heat and freezing assays, we fitted logistic curves with a Weibull function on the change in *F*_V_/*F*_M_ with temperature for each plant replicate using the *fitplc* package (Duursma and Choat 2017). We modified the *K*_max_ argument so that it corresponded to the average control temperature *F*_V_/*F*_M_ value for each plant replicate.

We used simple linear regressions to examine the relationships between volumetric soil moisture and water potential. The *Ψ*_pd_ and *Ψ*_md_ were converted to positive values and log transformed to normalise the distribution of the residuals. To account for repeated measurements we applied and evaluated linear mixed effects models, using *lmer* in the *‘lme4’* package (Bates et al. 2015) for the leaf temperature data that were collected during the glasshouse heat stress events. Leaf temperatures measured during the day (9:00–18:00) and at night (18:00–6:00) were treated separately to reflect the change in day/night conditions in the temperature-controlled glasshouse. Leaf temperature models included watering treatment (high vs low) as the categorical fixed effect and leaf replicate (*n* = 4) as the random effect (intercept only). Two-way (watering treatment × heat stress exposure) analysis of variance (ANOVA) tests were also conducted on plant replicate data (*n* = 5 per watering treatment/ heat stress combination) to assess watering treatment and heat stress exposure effects on heat tolerance (TT^heat^), freezing tolerance (TT^frost^), total dry biomass (TDB), root:shoot ratio (*R*/*S*), pre-dawn leaf water potential (*Ψ*_pd_), and midday leaf water potential (*Ψ*_md_). When differences between the main effects were significant, multiple comparisons of means (post-hoc Tukey’s honestly significant difference test) were carried out. Data were checked for normality before analyses.

## Results

### Low water availability increased leaf temperature

**Fig. 2:**
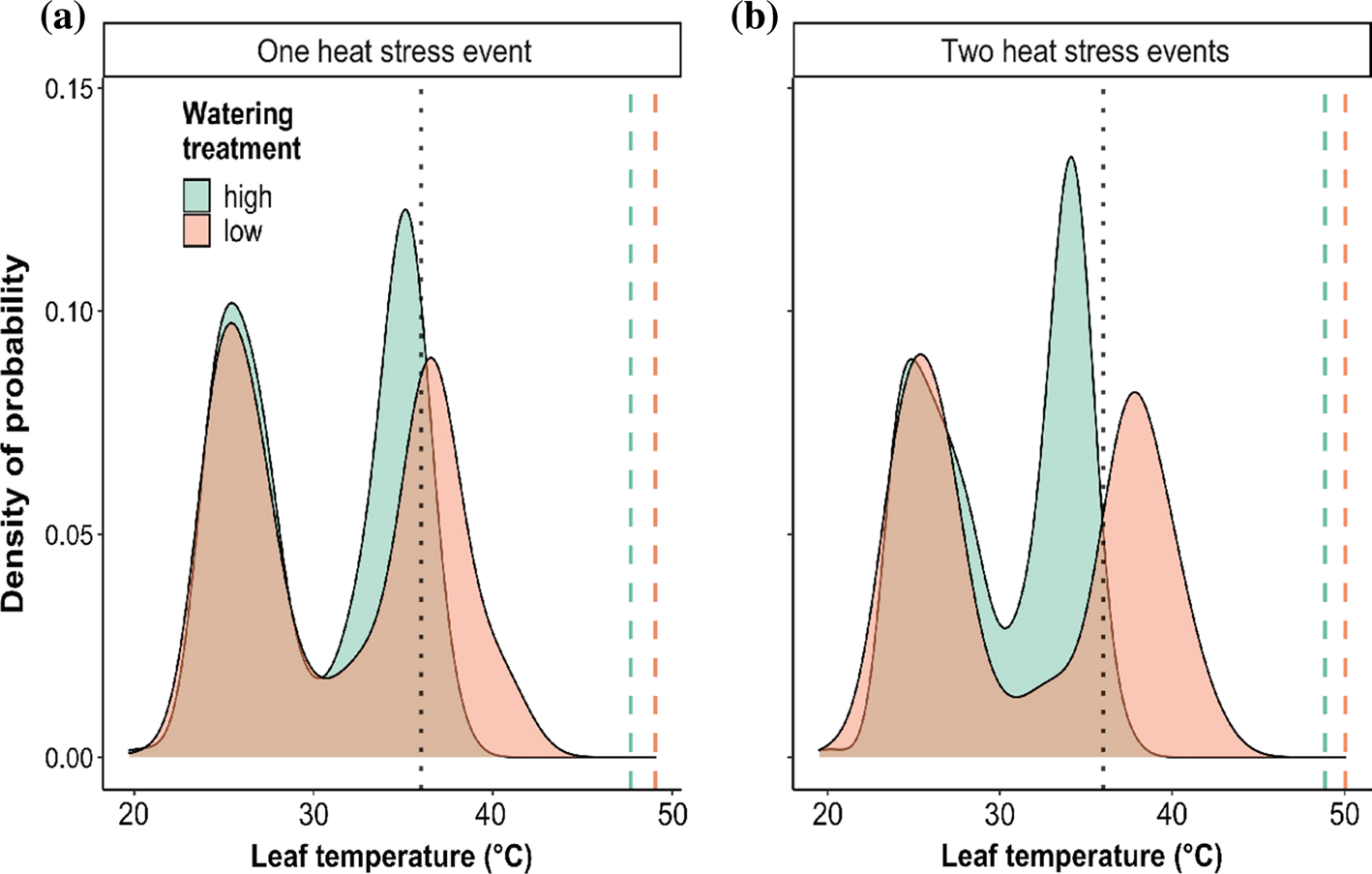
Density distributions for maximum leaf temperatures recorded amongst plants in high (*green*) and low (*orange*) watering treatments during exposure to **a** single heat stress event and to **b** a second heat stress event. Leaf temperature maxima reflect the maximum value of recorded leaves amongst plant replicates (*n* = 4). The *black vertical dotted line* indicates the maximum air temperature recorded during the glasshouse heat stress period. Watering treatment ‘high’ refers to plants watered to 100% PC and ‘low’ refers to plants held at 60% PC. Note that leaf temperatures did not exceed the mean TT^heat^ values estimated for plants in the high watering treatment (*green vertical dotted line*) or low watering treatment (*orange vertical dotted line*)

During exposure to one glasshouse heat stress, daytime leaf temperatures were 2.7 °C higher (*β* = 2.7, 95% CI = 2.60, 2.80) amongst plants in the low watering treatment compared to plants in the high watering treatment (intercept = 33.23) (Fig. 2). At night, leaf temperatures were significantly, but only slightly higher (0.5 °C) in the low watering treatment (*β* = 0.55, 95% CI = 0.39, 0.94) compared to those in the high watering treatment (intercept = 26.21) (Fig. 2).

During exposure to a second glasshouse heat stress, daytime leaf temperatures amongst plants in the low watering treatment were 3.65 °C higher (*β* = 3.65, 95% CI = 3.51, 3.78) than plants in the high watering treatment (intercept = 33.51) (Fig. 2). At night, leaf temperatures were also significantly higher amongst plants in the low watering treatment (*β* = 0.62, 95% CI 0.44, 0.80) compared to plants in the high watering treatment (intercept = 26.26) (Fig. 2).

### Leaf water potential decreased with water and heat stress

Following the glasshouse heat stress, the average pre-dawn volumetric soil moisture was 46.06% ± 1.05 SE in the high watering treatment (100% PC) compared to 14.49% ± 0.72 SE amongst plants in the low watering treatment (60% PC). Soil moisture declined to 40.84% ± 0.78 SE and 11.95% ± 0.78 SE by midday in the high and low watering treatments, respectively. Significant positive relationships were exhibited between volumetric soil moisture and both *Ψ*_pd_ (Supplementary materials Fig. S4; Table S1: *r*^2^ = 0.51, *F* = 29.43, *P* < 0.001) and *Ψ*_md_ (Supplementary materials Fig. S4, Table S2: *r*^2^ = 0.55, *F* = 34.35, *P* < 0.001).

**Fig. 3:**
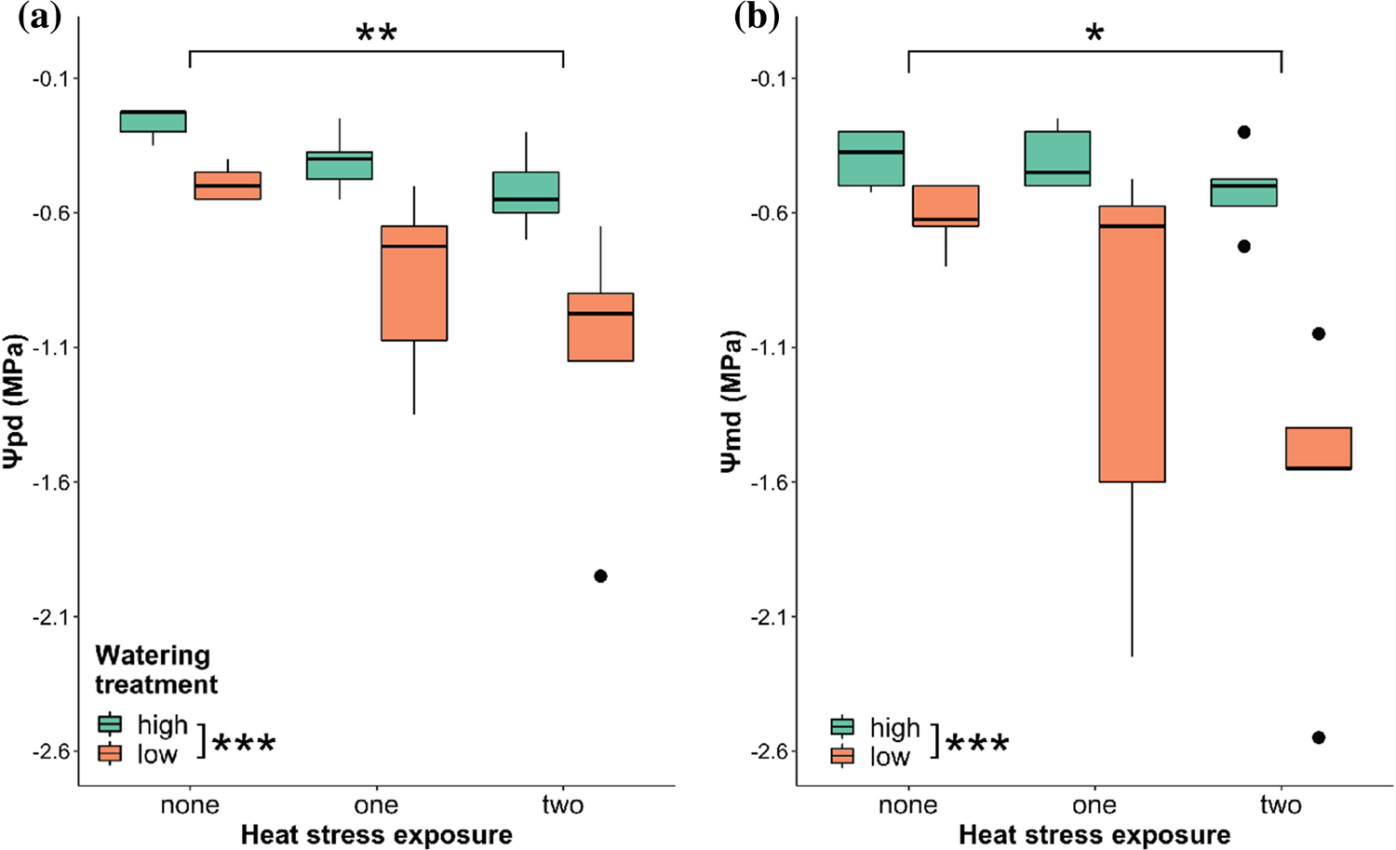
Response of **a** predawn water potential (*Ψ*_pd_) and **b** midday water potential (*Ψ*_md_) following the glasshouse heat stress, where asterisks indicate significant differences between heat stress exposure groups or watering treatment groups (significance codes: *P* < 0.001***, *P* < 0.01**, *P* < 0.05*). For the two watering treatments, ‘high’, refers to plants watered to 100% PC and ‘low’*,* refers to plants held at 60% PC. The statistical effect of both heat stress exposure groups and watering treatments are given in Table 1. *Black dots* show outliers

There was no interaction between the effects of watering treatment and heat stress exposure in *Ψ*_pd_ and *Ψ*_md_ (Table 1). Watering treatment, however, did result in a significant difference between the *Ψ*_pd_ (*F* = 20.075, *df* = 1,24, *P* < 0.001) and *Ψ*_md_ (*F* = 20.877, *df* = 1,24, *P* < 0.001) in *P. hothamensis* leaf blades (Fig. 3). The low watering treatment caused *Ψ*_pd_ and *Ψ*_md_ to decline an average of − 0.83 MPa ± 0.11 SE and − 1.12 MPa ± 0.17 SE respectively, whereas *Ψ*_pd_ and *Ψ*_md_ remained higher (i.e., closer to zero or less negative) amongst plants in the high watering treatment with averages of − 0.40 MPa ± 0.04 SE and − 0.44 MPa ± 0.03 SE, respectively.

**Table 1:** *F*-values and significance levels for factorial ANOVAs of the independent variables watering treatment (W), heat stress exposure (HS) and their combination on the physiological and growth properties in plants after glasshouse heat stress events

Sampling period	Variables	TT^heat^	TT^frost^	TDB	R/S	*Ψ*_pd_	*Ψ*_md_
After heat glasshouse heat stress	W	12.723**	15.489***	22.395***	2.415	20.075***	20.877***
	HS	17.752***	2.006	8.416***	2.684	7.339**	4.788*
	W × HS	0.049	1.664	5.748***	0.307	1.342	3.022
After six-week recovery	W	4.814*	0.015	1.353	0.940	NA	NA
	HS	2.389	0.532	0.468	0.048	NA	NA
	W × HS	0.7612	8.77	0.906	0.391	NA	NA

Heat tolerance °C (TT^heat^), freezing tolerance °C (TT^frost^), total dry biomass (g) (TDB), root:shoot (R/S), predawn water potential MPa (Ψ_pd_), and midday water potential MPa (*Ψ*_md_)

Significance codes: *P* < 0.001***, *P* < 0.01**, *P* < 0.05*

Heat stress exposure also produced a significant difference between *Ψ*_pd_ (*F* = 7.339, *df* = 2,24, *P* < 0.01) and *Ψ*_md_ (*F* = 4.788, *df* = 2,24, *P* < 0.05) (Table 1). Post-hoc comparisons indicated that compared to the control group (− 0.38 MPa ± 0.04), *Ψ*_pd_ declined significantly when plants were exposed to two (− 0.82 MPa ± 0.15 SE, *P* < 0.01) heat stress events. Similarly, a decline in *Ψ*_md_ was evident when plants were exposed to two heat stress events (− 1.07 MPa ± 0.22 SE, *P* < 0.05) compared to the control group (− 0.51, MPa ± 0.05 SE) (Fig. 3).

### Heat tolerance increased with water and heat stress

**Fig. 4:**
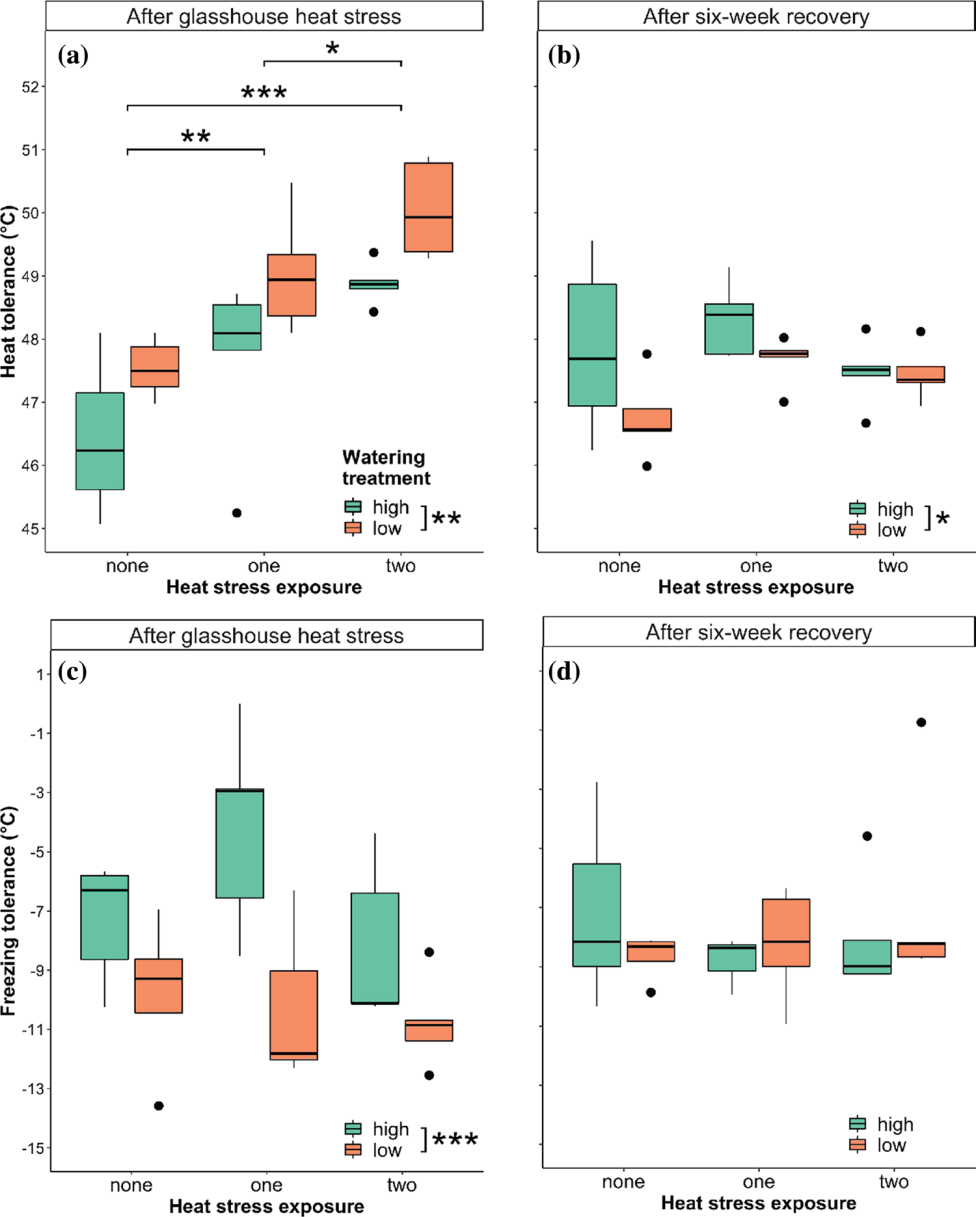
Response of **a**, **b** heat tolerance, and **c**, **d** freezing tolerance in plants determined **a**, **c** immediately after the glasshouse heat stress and **b**, **d** following a six-week recovery period, where asterisks indicate significant differences between heat stress exposure groups and watering treatment groups (significance codes: *P* < 0.001***, *P* < 0.01**, *P* < 0.05*). For the two watering treatments, ‘high’, refers to plants watered to 100% PC and ‘low’*,* refers to plants held at 60% PC. The statistical effect of both heat stress exposure groups and watering treatments are given in Table 1. Black dots show outliers

No significant interaction was detected between the effects of watering treatment and heat stress exposure on TT^heat^ in *P. hothamensis* (Table 1). There were, however, significant main effects of watering treatment (*F* = 12.723, *df* = 1,24, *P* < 0.01) and heat stress exposure (*F* = 17.752, *df* = 2,24, *P* < 0.001). Post-hoc comparisons indicated that TT^heat^ was significantly (*P*< 0.001) higher amongst plants in the low watering treatment (48.88 °C ± 1.27 SE) compared to those in the high watering treatment (47.67 °C ± 1.44 SE) (Fig. 3). TT^heat^ also significantly increased (*P* < 0.01) in plants after exposure to one heat stress event (*P* < 0.01; 48.36 °C ± 1.34 SE) and two heat stress events (*P* < 0.05; 49.47 °C ± 0.83 SE) compared to plants with no heat stress exposure (46.99 °C ± 1.03 SE) (Fig. 4). There was no interaction between the effects of watering treatment and heat stress exposure on TT^heat^ following the six week recovery period (Fig. 4; Table 1). However, there was a main effect of watering treatment (*F* = 4.814, *df* = 1,24, *P* < 0.05), with slightly higher TT^heat^ evident in plants in the high watering treatment (47.88 °C ± 0.24 SE) compared to plants in the low watering treatment (47.29 °C ± 0.16 SE) (Fig. 4).

### Freezing tolerance increased with water stress

There was no interaction between the effects of watering treatment and heat stress exposure on TT^frost^ in *P. hothamensis* following the glasshouse heat stress (Table 1). Post-hoc comparisons revealed no significant main effect of heat stress exposure on TT^frost^, although there was a significant main effect of watering treatment (*F* = 15.489, *df* = 1,23; *P* < 0.001) with higher TT^frost^ among plants in the low watering treatment (− 10.32 °C ± 2.17 SE) compared to those in the high watering treatment (− 6.58 ± 3.12 SE) (Fig. 4). Neither water treatment, heat stress exposure, nor their interaction, affected TT^frost^ of *P. hothamensis* plants following the six-week recovery period (Table 1; Fig. 4).

### Heat and water stress reduced plant growth

**Fig. 5:**
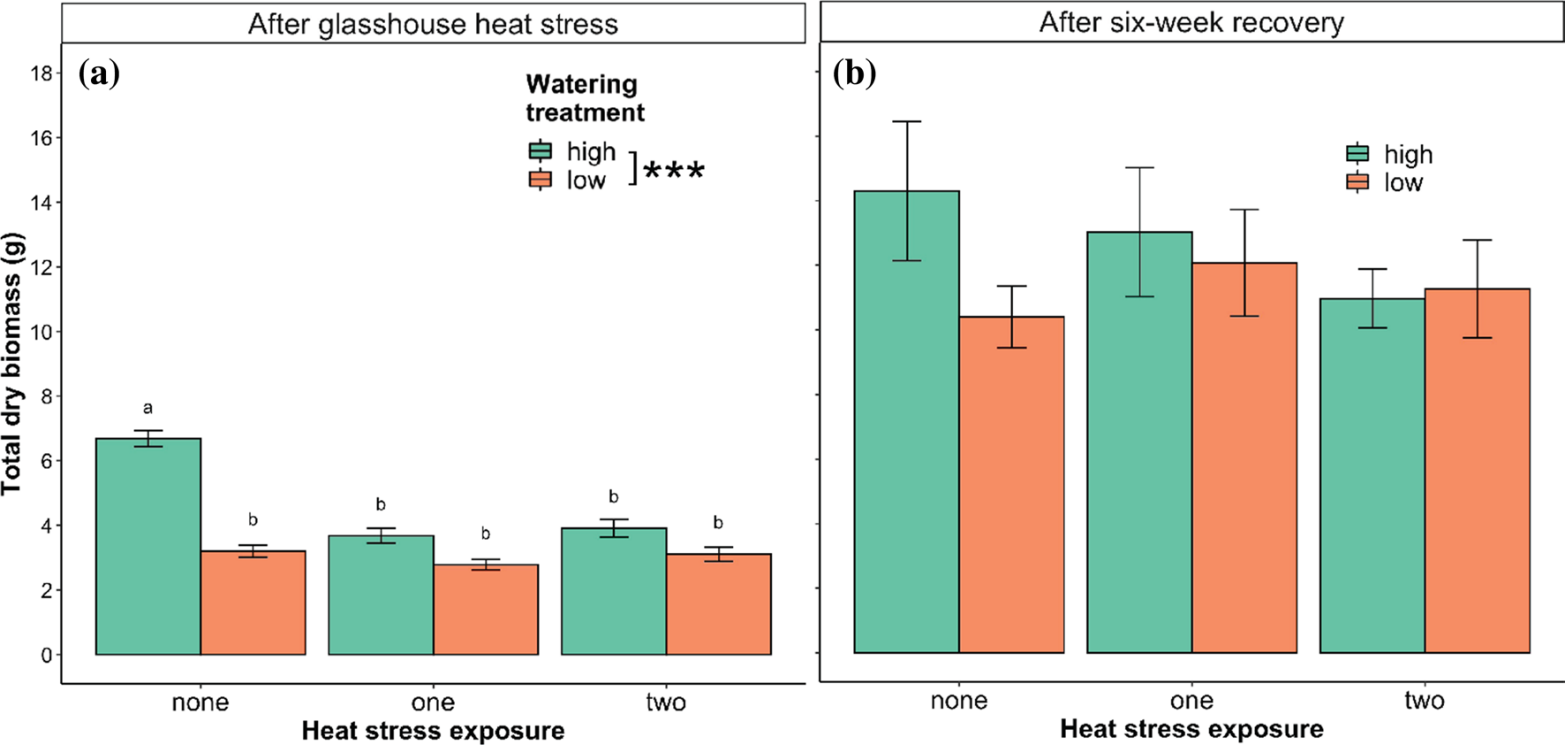
Mean ± SE total dry biomass (TDB) in plants sampled after **a** the glasshouse heat stress and **b** after the six-week recovery period. *Different letters* indicate significant differences according to Tukey’s HSD post-hoc comparison, *n* = 5 plant replicates. Columns not connected by the same letter represent significant differences. Watering treatment ‘high’ refers to plants watered to 100% PC and ‘low’ refers to plants held at 60% PC

Watering treatment and heat stress exposure interacted to significantly affect the total dry biomass (TDB) of *P. hothamensis* plants measured after the glasshouse heat stress (*F* = 23.52, *df* = 2,24; *P* < 0.001) (Fig. 5, Table 1). TDB was highest among plants in the high watering treatment with no heat stress exposure (6.68 g ± 0.55 SD) and reduced significantly in every other watering treatment and heat stress exposure combination, falling between 2.79 g and 3.91 g (Table 2). Watering and heat stress treatments had no effect, however, after the six-week recovery period where mean TDB across all treatment groups was 12 g (Fig. 5, Table 2). No effects of watering treatment or heat stress exposure on root:shoot of *P. hothamensis* were observed after the glasshouse heat stress or following the six-week recovery period (Table 1).

**Table 2:** Averages (mean ± SD) of heat tolerance °C (TT^heat^), freezing tolerance °C (TT^frost^), total dry biomass (g) (TDB), root:shoot (R/S), predawn water potential MPa (Ψ_pd_), and midday water potential MPa (Ψ_md_) amongst plants in watering and heat stress exposure treatments at two different sampling times: after glasshouse heat stress events, and after a six-week recovery period

	Watering treatment	Heat stress exposure	Measurement					
			TT^heat^	TT^frost^	TDB	*R*/*S*	*Ψ*_pd_	*Ψ*_md_
After glasshouse heat stress events	High	none	46.43 ± 1.21	− 7.33 ± 2.03	6.68 ± 0.55	0.51 ± 0.06	− 0.27 ± 0.06	− 0.40 ± 0.11
		One	47.68 ± 1.41	− 4.18 ± 3.36	3.69 ± 0.51	0.62 ± 0.18	− 0.41 ± 0.11	− 0.40 ± 0.12
		Two	48.88 ± 0.34	− 8.24 ± 2.71	3.91 ± 0.60	0.66 ± 0.17	− 0.52 ± 0.15	− 0.52 ± 0.15
	Low	None	47.54 ± 0.46	− 9.78 ± 2.77^i^	3.20 ± 0.43	0.39 ± 0.14	− 0.49 ± 0.07	− 0.62 ± 0.12
		One	49.04 ± 0.94	− 10.30 ± 2.59	2.79 ± 0.37	0.59 ± 0.15	− 0.86 ± 0.35	− 1.11 ± 0.78
		Two	50.05 ± 0.76	− 10.78 ± 1.52	3.10 ± 0.48	0.53 ± 0.24	− 1.13 ± 0.49	− 1.62 ± 0.56
After six − week recovery	High	None	47.86 ± 1.36	− 7.15 ± 3.02	14.30 ± 4.83	0.74 ± 0.41	n.d	n.d
		One	48.32 ± 0.59	− 8.76 ± 0.76	13.02 ± 4.45	0.67 ± 0.30	n.d	n.d
		Two	47.47 ± 0.53	− 8.03 ± 1.98	10.97 ± 2.04	0.65 ± 0.42	n.d	n.d
	Low	None	46.75 ± 0.65	− 8.65 ± 0.82^i^	10.40 ± 2.13	0.47 ± 0.18	n.d	n.d
		One	47.67 ± 0.39	− 8.23 ± 1.85	12.07 ± 3.69	0.63 ± 0.40	n.d	n.d
		Two	47.46 ± 0.43	− 6.90 ± 3.45	11.26 ± 3.39	0.60 ± 0.24	n.d	n.d

All measurements with *n* = 5; values with superscript ^i^ indicates *n* = 4

*n.d* not determined

## Discussion

Ongoing changes in global climate are expected to drive increases in the frequency and severity of extreme conditions such as droughts and heatwaves, with significant consequences for the physiological stress responses of alpine plants. Here, we showed water stress alters thermal tolerance in the common alpine grass, *P. hothamensis,* where physiological adjustments to low watering, indicated by more negative pre-dawn and midday water potentials, were also accompanied by shifts to higher freezing tolerance (improved tolerance to more negative temperatures) and to higher heat tolerance (improved tolerance to higher temperatures). Shifts to higher heat tolerance were also evident with exposure to heat stress applied in the glasshouse, though freezing tolerance was not affected. The combined heat and water stress appeared to also have a cumulative effect on photosynthetic heat tolerance. The co-occurrence of stress factors is often demonstrated as having ongoing effects on growth (Yang et al. 2005; De Boeck et al. 2016; Bachofen et al. 2019). Our results indicate that, while *P. hothamensis* growth was initially inhibited by heat stress or water stress, the effects of these stressors appeared to be short-lived, with little differences in biomass and thermal tolerance following the six-week recovery period.

Photosynthetic heat tolerance is significantly influenced by temperature conditions. In alpine ecosystems, temperature and solar radiation can change quickly throughout the day, exposing plants to rapid fluctuations in temperature (Körner and Hiltbrunner 2018). For example, the heat tolerance in an alpine cushion plant began to increase at temperatures above 30 °C (Neuner et al. 2000), and rapid acclimation to hotter temperatures has been demonstrated in the field with diurnal adjustments of heat tolerance of up to 9.5 °C (Buchner and Neuner, 2003). The nature of prior exposure to hot temperatures (e.g., frequency, duration, and magnitude) likely also affects acclimation of heat tolerance. In this study, plants exposed to two, rather than one heat stress day achieved higher photosynthetic heat tolerance even though plants were given one cool day in between heat stress events to recover. This indicates that while acclimation to hotter temperatures can improve photosynthetic heat tolerance throughout the course of a day, de-acclimation (i.e., the loss of heat tolerance) may require the removal of high-temperature stress over a longer period. The retention of high heat tolerance following amelioration of high-temperature conditions is likely to benefit plants growing in alpine environments characterised by rapid fluctuations in temperature by widening the margin between temperature maxima and thermal tolerance thresholds. Further improvements in heat tolerance may also be possible with ongoing exposure to heat stress, though previous research has indicated that upper thermal tolerance is more physiologically constrained than freezing tolerance as high-temperature limits tend to vary less than low-temperature limits (Lancaster and Humphreys 2020).

Plants in the low watering treatment exhibited more negative Ψ_pd_ and Ψ_md_ compared to well-watered plants, a trend exacerbated by exposure to two heat stress days. Experiencing prolonged or combined heat and water stress may place plants under increased risk of hydraulic failure. Water stress is also known to result in higher heat tolerance, in some instances to a greater degree than elevated growing temperature (Ghouil et al. 2003). Water stress can induce several physiological changes in plants, including the closure of stomata which can limit the difference in water potential between the soil and leaves (Martínez-Vilalta and Garcia-Forner 2017). Stomatal closure can also reduce the capacity of plants to cool their leaves via transpiration. Hence water-stressed plants can have higher maximum leaf temperatures (Ladjal et al. 2000), as was seen in *P. hothamensis* plants in the low watering treatment during heat stress days. Photosynthetic heat tolerance may be driven by higher maximum leaf temperature values (Curtis et al. 2019; Perez and Feeley 2020). Higher heat tolerance driven by leaf temperature may allow plants to maintain adequate safety margins between temperature maxima and thermal limits. Acquired high heat tolerance evident in *P. hothamensis* following water stress or heatwave exposure also coincided with significant reductions in biomass. Heat and water stress can inhibit carbohydrate metabolism with consequences such as reduced plant growth and development (Kaushal et al. 2013). The combined stressors did not appear to have a cumulative effect on biomass, however, and initial differences were also not retained after the six-week recovery period, indicating a fast growth capacity in *P. hothamensis* following the removal of water and high-temperature stress.

Adaptations to frost are common and widespread among the Poaceae, though species that experience longer frost periods or colder temperatures in their geographic ranges typically have the highest tolerance to freezing conditions (Humphreys and Linder 2013; Schubert et al. 2020). To tolerate frost, grasses rely on cold acclimation, and frost-resistant grasses have developed various mechanisms that prevent the formation of large ice crystals or limit ice nucleation that could physically damage plant cells (Schubert et al. 2020). De-acclimation, and the associated loss of freezing tolerance, typically follow warmer temperatures and a resumption of growth in spring (Franklin et al. 2014). Despite evidence that exposure to heat can affect subsequent freezing tolerance in some species (Lafuente et al. 1991; Fu et al. 1998), this was not the case in *P. hothamensis* following exposure to one or two heat stress days. It is possible that during the growing season, *P. hothamensis* retains a baseline freezing resistance which affords protection from episodic frosts that can occur year-round in the Australian alpine zone. Indeed, while alpine plants typically lose freezing tolerance with the onset of the growing season, freezing tolerance thresholds that are far below low-temperature minima have been reported in grasses during summer in the alpine zone (Bannister 2005). It is also possible that plants may lose freezing tolerance in response to moderate but longer increases in ambient growing temperatures (Sierra-Almeida and Cavieres 2010), as opposed to the short-term heat stress applied in this study. We did, however, find a significant shift to higher freezing tolerance amongst plants in the low watering treatment, corroborating previous research and indicating strong links between tolerance to water stress and frost (Sierra-Almeida et al. 2016). Improved freezing tolerance in water-stressed plants is often attributed to the overlap in physiological changes linked to both stressors (Kong and Henry 2019b).

Shifts in heat and freezing tolerance driven by exposure to water stress or heat stress appeared to be transient, with no effect present after a six-week growing period when plants were returned to more benign growing conditions. Leaf material used to estimate thermal tolerance following the recovery period, however, was likely derived from new growth, and photosynthetic thermotolerance has previously been positively correlated with leaf life span and leaf age (Zhang et al. 2012; Ruocco et al. 2019). As such, it is likely that reduced photosynthetic heat and freezing tolerance following the six-week recovery reflected the more benign conditions experienced by new leaves. Alternatively, a third heat stress event could see a continued increase of heat tolerance to higher levels than achieved by the second heat stress event. Stress memory has also previously been shown to manifest as fitness improvements (i.e., through improved flowering and seed set) following subsequent extreme temperature events (Bruce et al. 2007) which can persist through generations (Whittle et al. 2009). The importance of temporal stability and heritability of stress memory may emerge as extreme climatic events become more frequent under climate change and deserves further attention. Initial differences seen in biomass accumulation were also not retained after the six-week recovery period, indicating a fast growth capacity in *P. hothamensis* following the removal of water and high-temperature stress. However, a high capacity to recover after the water stress treatment does not necessarily indicate drought tolerance in this species as root:shoot, a trait considered to increase in response to water stress in drought-resistant species (Couso and Fernández 2012), was unresponsive to water stress. Plants that allocate a higher proportion to root than to shoot production can improve access to water and nutrients and reduce the detrimental effects of water stress (Poorter and Nagel 2000). The low watering treatment was maintained for a three-week period, however, rain-free periods in the Australian Alps are expected to increase in duration as the incidence of drought increases with ongoing climate change (Hennessy et al. 2008). In a previous study, *P. hothamensis* was found to be sensitive to water stress, with mortality linked to low water availability in the Australian alpine zone following periods of up to 40 days without a significant rain event during summer (Griffin and Hoffmann 2012).

In conclusion, our results indicate that *P. hothamensis* has a high capacity to acclimate to combined water and heat stress, which demonstrates a robust and ecologically important response to the fluctuating and co-occurring environmental stresses common during the growing season in the Australian Alps. Rapid acclimation of photosynthetic heat tolerance to combined temperature and water stress may improve survival outcomes under a hotter, drier climate. Insensitivity of photosynthetic freezing tolerance to heat stress events in *P. hothamensis* also ensures the maintenance of a high capacity to tolerate episodic frosts that can occur during summer. Moreover, a high capacity for rapid growth may ensure the persistence of the species following stress events. The extent to which further improvements in thermal tolerance can occur under chronic or frequently repeated stress events, however, is uncertain, and the capacity for plants to recover from the heat and/or water stress likely depends on the nature of the stress factors. Future research should focus on the duration, timing, magnitude, and frequency of stress events, as responses will largely depend on it.

## Supplementary Information

Below is the link to the electronic supplementary material.

Supplementary file1 (DOCX 2287 KB)

## Data Availability

The authors confirm that data underlying the findings are fully available under the https://doi.org/10.6084/m9.figshare.20046191
