# Supplementary material for: Acclimation to water stress improves tolerance to heat and freezing in a common alpine grass
Source: Oecologia. 2022 Aug 17;199(4):831–43. doi: 10.1007/s00442-022-05245-1 (PMC9464112; doi:10.1007/s00442-022-05245-1)
Supplement: Supplementary file 1 — Supplementary file1 (DOCX 2287 KB) [file 442_2022_5245_MOESM1_ESM.docx]

**Acclimation to water stress improves tolerance to heat and freezing in a common alpine grass**

Emma E. Sumner^1*^, Virginia G. Williamson^1^, Roslyn M. Gleadow^2^, Tricia Wevill^1^ and Susanna E. Venn^1^

^1^Centre for Integrative Ecology, Deakin University, Burwood 3125, Australia
^2^School of Biological Sciences, Monash University, Clayton 3800, Australia

*Author for correspondence: [eesumner@deakin.edu.au](mailto:eesumner@deakin.edu.au)


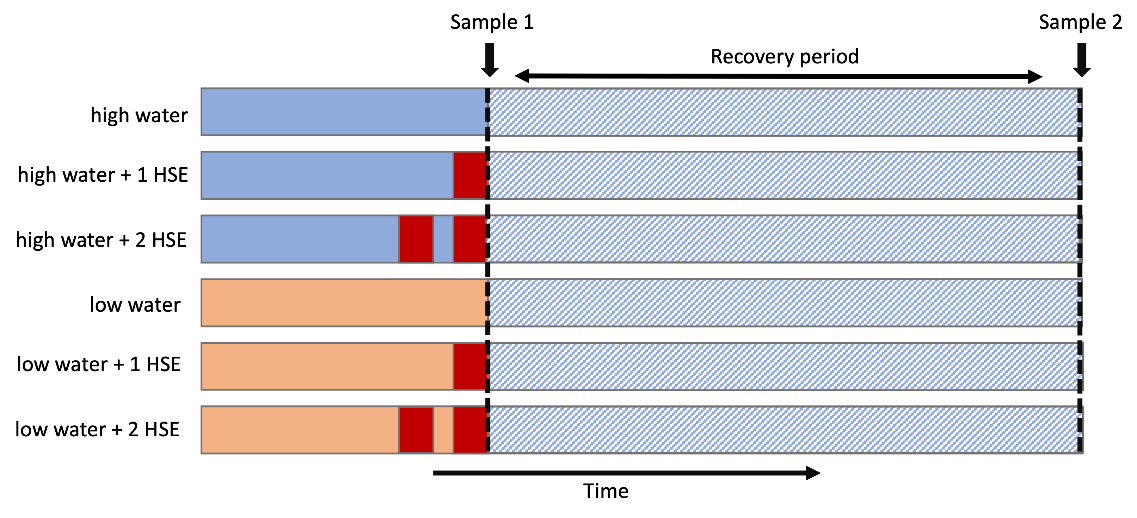


**Fig 1** Timeline of experiment. Plants were subjected to high (100% PC) (blue) or low (60% PC) (orange) watering regimes for three weeks and subsequently exposed to either one, two, or no heat stress events in the glasshouse (HSE indicated by red bars). Vertical dashed line indicates sample collection for heat and freezing assays following watering and heat stress event treatments and again following a six week recovery period.


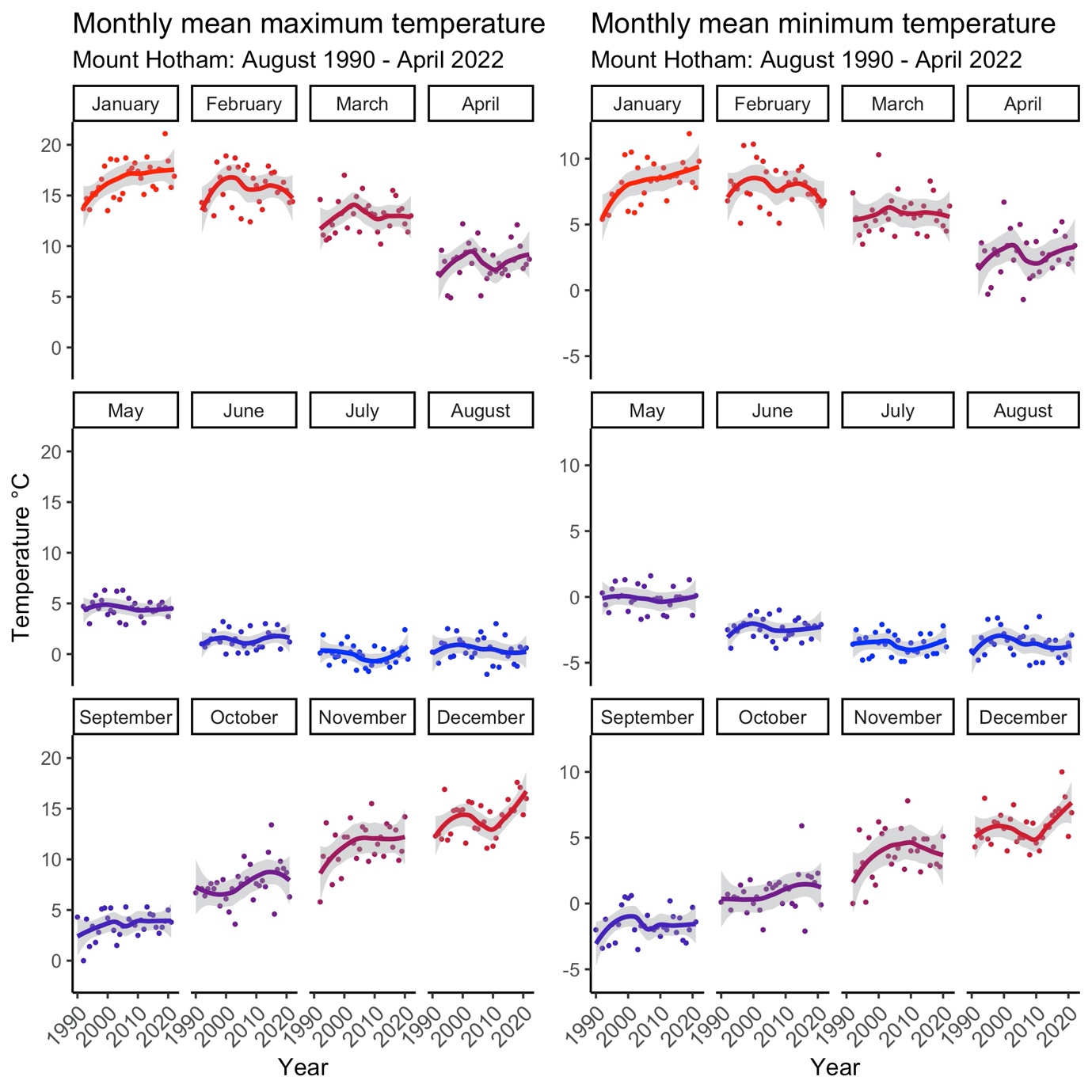


**Fig 2** Monthly mean (left panel) maximum and (right panel) minimum air temperatures reported for Mount Hotham weather station between August 1990 to April 2022: Bureau of Meteorology station number 83085, 1849 m asl, lat long: -36.98, 147.13 Grey shading indicates 95% CI.

**
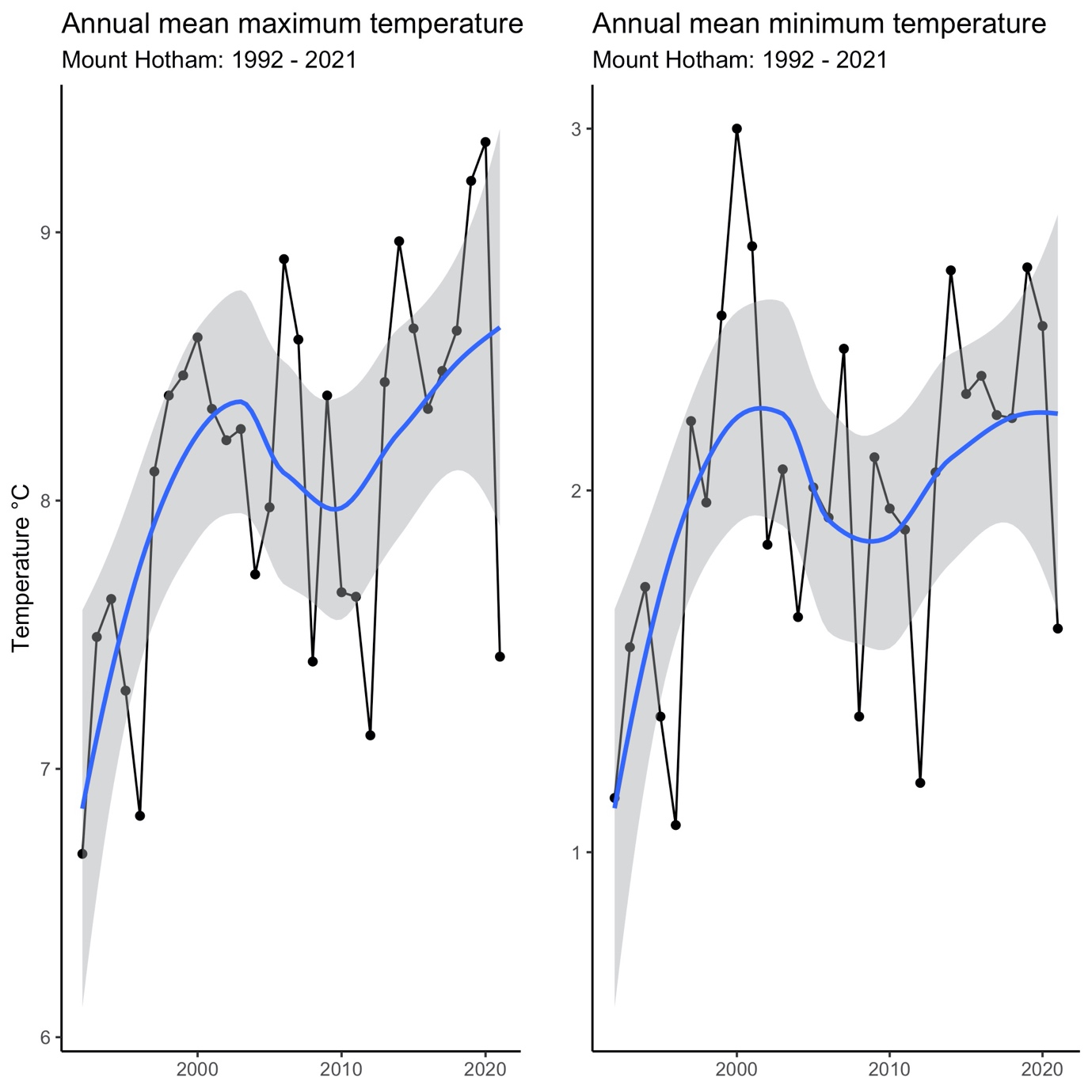
**

**Fig 3** Annual mean (left panel) maximum and (right panel) minimum temperature reported for Mount Hotham weather station between 1992 to 2021: Bureau of Meteorology station number 83085, 1849 m asl, lat long: -36.98, 147.13. Grey shading indicates 95% CI.

__

**Fig 4** Increase in Ψ_pd_ (a) and Ψ_md_ (b) with increasing volumetric soil moisture. Lines were fitted by linear regression. Colours indicate different watering treatments: green, high; orange, low. Note the use of log scale on the *y*-axes. Data for Ψ_pd_ and Ψ_md_ are pooled across experimental watering and heatwave treatments.

Table 1. Regression coefficients for predicting log Ψ_pd_ with soil moisture in *P. hothamensis*

| **Variable** | **Estimate** | **95% CI** | | **t value** | **p-value** | |
| --- | --- | --- | --- | --- | --- | --- |
| (Intercept) | 0.032414 | [-0.1007126, 0.165540211] | | 0.499 | | 0.622 |
| soil moisture | -0.010276 | [-0.0141562, -0.006396078] | | -5.425 | | 8.69e-06*** |
| Multiple R-squared: 0.5125 | | |  |  | |  |

Table 2. Regression coefficients for predicting log Ψ_md_ with soil moisture in *P. hothamensis*

| **Variable** | **Estimate** | **95% CI** | **t value** | | **p-value** |
| --- | --- | --- | --- | --- | --- |
| (Intercept) | 0.150292 | [0.01028407, 0.290300349] | 2.199 | | 0.0363 |
| soil moisture | -0.013251 | [-0.01788298, -0.008619761] | -5.861 | | 2.66e-06*** |
| Multiple R-squared: 0.5509 | |  | |  |  |
